# Mice Expressing *RHAG* and *RHD* Human Blood Group Genes

**DOI:** 10.1371/journal.pone.0080460

**Published:** 2013-11-18

**Authors:** Dominique Goossens, Nelly da Silva, Sylvain Metral, Ulrich Cortes, Isabelle Callebaut, Julien Picot, Isabelle Mouro-Chanteloup, Jean-Pierre Cartron

**Affiliations:** 1 Institut National de la Transfusion Sanguine, Paris, France; 2 Inserm UMR_S 665, Paris, France; 3 Université Paris Diderot, Sorbonne Paris Cité, UMR-S665, Paris, France; 4 IInstitut de Minéralogie et de Physique des milieux Condensés UMR 7590 CNRS, Université Pierre et Marie Curie, Paris, France; Nathan Kline Institute and New York University School of Medicine, United States of America

## Abstract

Anti-RhD prophylaxis of haemolytic disease of the fetus and newborn (HDFN) is highly effective, but as the suppressive mechanism remains uncertain, a mouse model would be of interest. Here we have generated transgenic mice expressing human RhAG and RhD erythrocyte membrane proteins in the presence and, for human RhAG, in the absence, of mouse Rhag. Human RhAG associates with mouse Rh but not mouse Rhag on red blood cells. In *Rhag* knockout mice transgenic for human *RHAG*, the mouse Rh protein is “rescued” (re-expressed), and co-immunoprecipitates with human RhAG, indicating the presence of hetero-complexes which associate mouse and human proteins. RhD antigen was expressed from a human *RHD* gene on a BAC or from *RHD* cDNA under control of β-globin regulatory elements. RhD was never observed alone, strongly indicative that its expression absolutely depends on the presence of transgenic human RhAG. This first expression of RhD in mice is an important step in the creation of a mouse model of RhD allo-immunisation and HDFN, in conjunction with the *Rh-Rhag* knockout mice we have developed previously.

## Introduction

The human Rh (Rhesus) blood group is of clinical interest due to its role in transfusion medicine, auto-immune anaemia and its implication in materno-fetal incompatibility and hemolytic disease of the fetus and newborn (HDFN) [1]. RhD is a highly immunogenic antigen and despite the effectiveness of RhD prophylaxis, materno-fetal immunisation due to RhD antigen, a cause of perinatal mortality and morbidity, is not completely eradicated [2-4]. Currently, monoclonal or recombinant anti-D are being tested for prophylaxis as alternatives to plasma derived polyclonal IgG anti-D. Although a number of models have been developed, the mechanism for anti-RhD suppression has as yet to be determined (reviewed in [5,6]). In this context, a transgenic mouse model of RhD antigen expression would be useful to study RhD allo-immunisation and HDFN.

Rh antigens are present in the erythrocyte membrane in an oligomeric association of two major components, Rh proteins (RhD and/or RhCcEe), and homologous Rh-associated glycoprotein (RhAG). The Rh complex also includes other proteins (ICAM-4/LW, CD47/IAP and glycophorin B), all linked by non-covalent bonds (reviewed in [7-10]). Rh-deficiency, a rare autosomal recessive disorder in man, is caused by mutations occurring either in the *RHAG* or *RH* locus. When the RhAG or the Rh subunit is absent, the Rh complex is missing or severely reduced [8,11]. 

Rh and RhAG proteins, which compose the core of the Rh complex, interact together within what is most likely to be a trimeric structure, based on crystal structure of RhCG [12], NeRh50 [13] and AmtB [14,15]. This is supported by transmission electron microscopy of the human homolog RhCG expressed in, and purified from, HEK293E cells [16]. A predictive model integrating the Rh-RhAG core complex as hetero-trimer within the AE1 multi-protein complex linking the membrane to the cytoskeleton has been proposed [17,18]. In mouse, the relationship between mRh and mRhag as well as other members of the Rh complex, differs somewhat from that in human erythrocytes. Notably, mRhag glycoprotein is less dependent on Rh in the mouse than in man: in knockout mice, mRhag is expressed in the absence of mRh, though at slightly reduced levels, but mRh cannot be expressed without mRhag [19]. CD47, accessory protein of the Rh complex in man, is independent of the complex in the mouse: CD47 is severely defective in 4.2-deficiency in man but not in mice [20,21], mouse CD47 lacks the cytoskeletal connectivity of the human protein [22] and Rh or Rhag knockout mice have no defect of mCD47 [19]. ICAM4 is associated with the Rh antigens and absent in Rh null phenotypes in mouse [19] as well as in man.

We have developed two transgenesis approaches to express the RhD antigen, using human genomic DNA in a bacterial artificial chromosome, and a β-globin LCR-promoter system [23]. It was established, both *in vitro* in mouse erythroleukemia MEL-C88 cell line, and *in vivo*, that mouse mRhag is not sufficient to allow expression of human RhD, which must be partnered with human RhAG (hRhAG) to reach the erythrocyte membrane. For the first time, mice expressing human RhD antigen on their erythrocyte membrane have been produced, and the various transgenic mice obtained provided new information on the Rh complex*.*

## Materials and Methods

### Antibodies

The following monoclonal antibodies were used

- for flow cytometry: rat anti-mouse CD44 (clone IM7), mouse CD47 (clone miap 301), and mouse CD71 (clone C2) from BD Biosciences Pharmingen; mouse monoclonal anti-hRhAG LA18.18 (a gift from Dr Von dem Borne, Amsterdam, The Netherlands), human anti-Band 3 (HIRO-58-Dib) cross-reacting with mouse Band 3 (a gift from Dr. M. Uchikawa, Japanese Red Cross Central Blood Center, Tokyo), anti-RhD human monoclonals H2D5D2F5 (INTS) and LOR15C9 (gift of Pr. A. Blancher, Toulouse, France) and LFB-R593 (gift of B. Fournes, LFB, France) and polyclonal anti-RhD Rhophylac (CSL Behring SA, France).
- for immunoblot: Mouse Rh and Rhag proteins (mRh and mRhag) were detected by rabbit polyclonal antibodies (INTS) raised against the C-terminal regions of, respectively, the human Rh polypeptide, cross-reactive with mRh (MPC8 [24]), and of mouse Rhag glycoprotein [21]. Mouse actin was detected by species cross-reactive anti-actin ab 3280 clone ACTN05 (C4) (Abcam, France). Anti-hRhAG LA18.18 and anti-RhD LOR15C9 were also used for immunoblot.

### pGSEL1 vectors, MEL-C88 cell line culture and transfection

**Figure 1:**
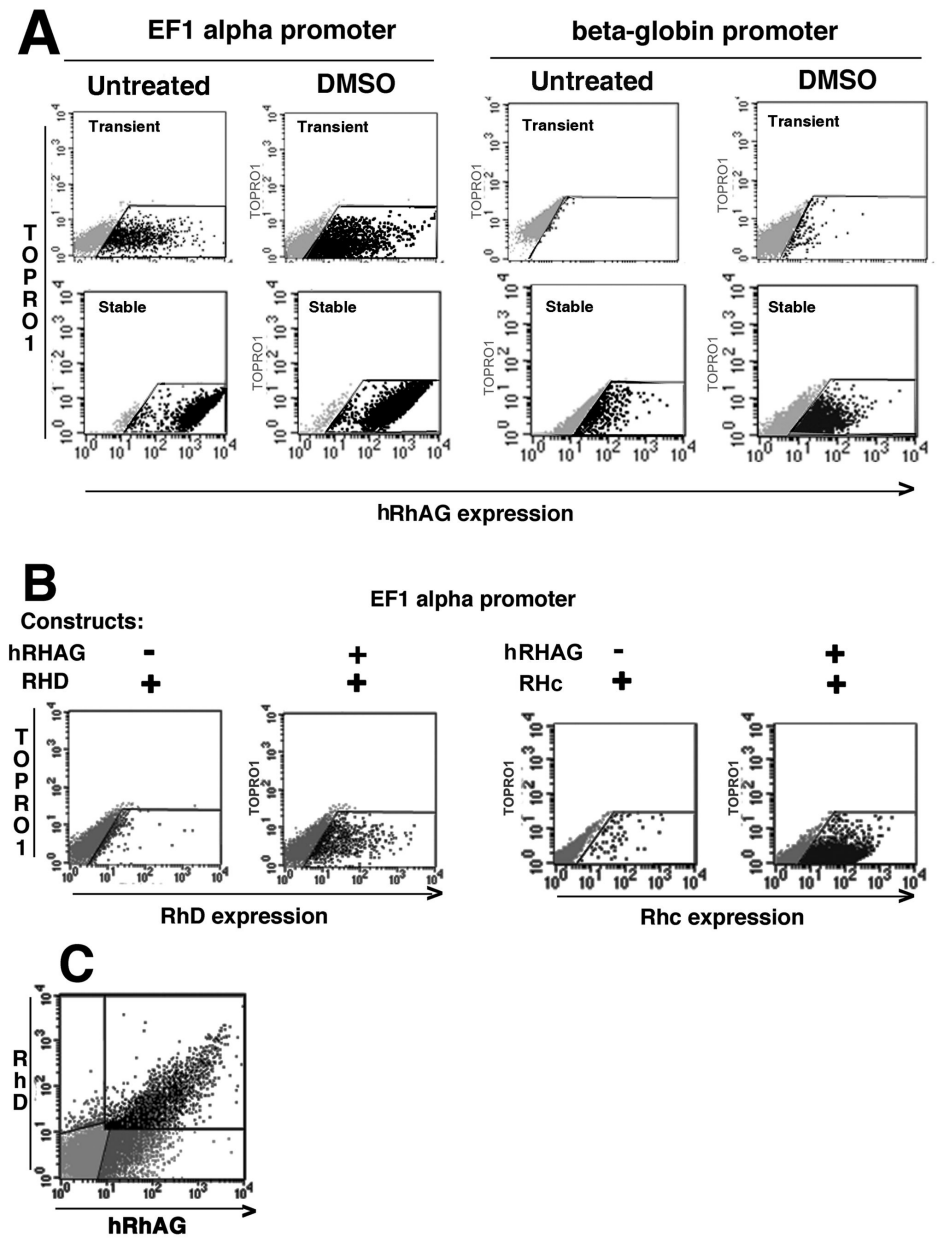
Flow cytometry analysis of hRhAG and hRh protein expression in MEL-C88 transfected cells. hRhAG can be expressed alone in a mouse erythroid context, but RhD or Rhce require the presence of hRhAG. (**A**) hRhAG expression in transient and stable transfections under the control of EF1-α promoter or of the β-globin promoter and enhancer. DMSO-induced erythroid differentiation increases expression levels. (**B**) RhD and Rhc expression in the absence or presence of hRhAG. (**C**) Dose dependence of RhD on hRhAG expression: double labeling experiments in transient co-transfection with EF1-α promoter.

Murine erythroleukemia line MEL-C88 [25] and pGSEL1 vector system [23] were a gift from M. Hollis (then at ICI Pharmaceuticals, UK). Human *RHAG, RHD and RHce* cDNAs (Figure 1A) were cloned into this system allowing protein expression under control of the β-globin LCR/promoter/enhancer.

MEL-C88 were cultured in RPMI 1640, 10% FCS (Fisher Bioblock). Erythroid differentiation was induced with 5 mM DMSO (Sigma). In initial experiments, differentiation was verified by expression of mCD44, mCD71, Band 3 and mCD47. Transfection was performed with Amaxa Nucleofector® (Lonza) on day 3 and hRhAG, RhD and Rhc expression tested by flow cytometry on days 4 to 6.

### pEF1-mycHis vector

Human *RHAG*, *RHD* and *RHce* cDNAs coding regions (as in Figure S1) with a eukaryotic Kozak sequence were cloned into pEF1-mycHis (Invitrogen).

### Transgenic mice

#### BAC selection and injection

BACs containing *RHD* and *RHAG* genes were identified by PCR screening of the bank of human BACs in pBELOBAC11 at the Centre d'Etude du Polymorphisme Humain (CEPH, Paris). Four BAC clones positive for the *RHD* gene and 8 for the *RHAG* gene were identified. These were further characterized for size and extension by pulse field gel electrophoresis (PFGE) and by PCR to ascertain the presence of complete gene and, for *RHD*, absence of *RHCE*. For injection, a BAC (CEPH n°H193H9) of the *RHD* group and a BAC (CEPH n°H0696H07) of the *RHAG* were selected (Table S1).

Transgenesis experiments were carried out at the Institut Pasteur, in the Laboratoire de Biologie du Développement (Pr Ch. Babinet), later at the Centre d’Ingénierie Génétique Murine (Dr F. Langa). BACs were micro-injected in circular form into fertilized C57Black6/SJL mouse oocytes. 

*Transgenesis of hRHAG and RHD under control of the β-globin LCR/promoter/enhancer* was carried out at the Service d'Expérimentation Animale de Transgénèse (SEAT) / UPS44 CNRS Villejuif (Drs.Goujet-Zalc and Martin). *RHAG* and *RHD* transgenes under control of the erythroid-specific β-globin promoter and LCR, excised from pEC3-pGSEL1 with *Aat*II to eliminate vector sequence, were purified and micro-injected into C57Bl/6 and B6/CBA fertilised oocytes.

Mouse breeding (including with Rhag^-/-^ mice [19]) and blood collection were carried out at SEAT /UPS44 CNRS Villejuif and at TAAM-CDTA /UPS44 CNRS (Orléans). All work was done in accordance with Policies and Directives for the Care and Use of Laboratory Animals of the CNRS, in compliance with French and European Union animal welfare policies. Blood samples were transferred to INTS for analysis. Personal licenses from the French Veterinary Services were N°75-862 for JPC, 75-1236 for DG, A 92-368 for SM. 

### Flow cytometry analysis

Surface antigen expression on cells or erythrocytes was determined by immunostaining, followed by flow cytometric analysis on a BD-FACSCanto™ II flow cytometer (Becton-Dickinson). When appropriate, cell viabilility was determined using a viability indicator such as TO-PRO®-1 iodide from Molecular Probes (abbreviated TOPRO1). Quantitative determination of surface antigens (antibody binding capacity) was done with QIFIKIT (DAKO). For intracellular epitope analysis (mRhag, mRh), erythrocytes were fixed in formaldehyde-glutaraldehyde, permeabilised with octyl-glucoside, blocked, immunostained and analysed by flow cytometry as described [26].

### Red cell membrane protein analysis

Membrane proteins from whole ghost lysates prepared by hypotonic lysis, were separated by SDS-PAGE (NuPage® 4-12% Bis-Tris, Invitrogen), transferred, and immunoblotted with relevant antibodies [27]. Revelation was with Amersham ECL™ Western Blotting System (GE Healthcare).

### Co-Immunoprecipitation

Anti-hRhAG LA18.18 was bound to ProteinGSepharose4 FastFlow beads (GE Healthcare). 100 µl of erythrocyte ghosts from TG-hRHAG 68.08 mice and wild type (WT) controls were solubilised in buffer (10 mM Tris/HCl pH7.4,150 mM NaCl, 0.5 mM CaCl2, 0.5 mM MgCl2, 0.5 mM PMSF + protease inhibitor 1x) containing 1% Triton X100. Solubilised membranes were pre-cleared on uncoupled beads 2h at 4°C, incubated overnight at 4°C with LA18.18-coupled beads while rotating, then washed 3 times in solubilisation buffer. Bound protein was eluted 40 min at room temperature in Laemmli buffer 1x and analysed by SDS-PAGE and immunoblot with specific antibodies.

### Quantitative Real-time PCR

Total RNA was isolated from whole blood of WT , BAC1 or BAC1 x 68.08 transgenic mice, using the High Pure RNA Isolation kit (Roche). No lysis of erythrocytes was performed to ensure reticulocytes were retained. cDNA was synthesized by reverse transcription-PCR (RT-PCR) using the SuperScript® VILO™ cDNA Synthesis Kit (Life Technologies). 

Quantitative real-time PCR was performed on the Applied Biosystems 7300 real-time PCR system and TaqMan® Endogenous Control mouse transferrin receptor-Mm00441941 (noted mTfR1) in duplex with TaqMan® gene expression assays (Life Technologies), with exon-spanning probes, for endogenous mouse Rh (Rhd mouse Mm00456910-m1), or transgenic human RhD (RhD/CE human Hs00414315-m1). Assay specificity was verified with the following negative controls: non-transfected mouse samples for *RHD* and mRh knockout samples for mouse *Rh*. Amplification efficiencies (E) were comparable for the three primer pairs (1.96-2). All samples were measured in triplicate, and two reverse transcriptions were tested. Standard curves for control and genes of interest were included in each experiment.No template controls and No RT controls were included in each assay, and amplification and detection were performed under standard conditions as recommended by manufacturer. The fluorescent signal intensities were recorded and analysed during PCR amplification using the Sequence Detection Software (SDS) software (Applied Biosystems). Efficiency corrected relative expression ratios for gene of interest (GOI) to mTfR1 were calculated as R=(E_target_) ^ΔCp target (control-sample^)/ (E_reference_) ^ΔCp reference (control-sample)^, with pooled cDNA from GOI positive mice as controls.

### NH_3_ and CH_3_NH_2_ uptake by resealed red cell ghosts

#### Resealed ghost preparations and diameter measurements

For stopped-flow analysis, erythrocyte ghosts were prepared as described [28]. All steps except resealing (37°C) were carried out at 4°C. 200 µl of blood were washed three times in PBS and resuspended in 20ml hypotonic lysis buffer (3.5 mM K_2_SO_4_, 10 mM Hepes/KOH, pH 7.2) for 40 min on ice followed by resealing for 1 hour in resealing buffer (50 mM K_2_SO_4_ 10mM Hepes/KOH, pH 7.2, 1 mM MgSO_4_) containing 0.15 mM pyranine (1-hydroxypyrene-3,6,8-trisulfonic acid, Sigma-Aldrich), a fluorescent pH sensitive dye. After three washes in the incubation buffer (50 mM K_2_SO_4_ 10 mM Hepes/KOH, pH 7.2), ghosts were kept on ice before assay in the same buffer. 

#### Stopped-flow assays

Stopped-flow experiments were performed at 15°C, as previously described [28]. Resealed ghost pellets were diluted at 1–2% cytocrit in the incubation medium (50 mM K_2_SO_4_ 10 mM Hepes/KOH, pH 7.2) and mixed (vol/vol) with ammonium buffer (40 mM K_2_SO_4_ 10mM (NH_4_)_2_SO_4_ 10 mM Hepes/KOH, pH 7.2) in the stopped-flow instrument (SFM400, Bio-Logic, Grenoble, France), generating an inwardly directed 10 meq NH_4_^+^ gradient. Data from six to eight time-courses were averaged and fitted to mono-exponential function using the simplex procedure of the Biokine software (Bio-logic). The excitation wavelength was 465 nm and emitted light was filtered with a 520 nm cut-off filter. The intracellular pH-dependent fluorescence changes were followed and analysed, as fluorescence increase corresponded to pH elevation. Over the pH range used (6.8–7.8) the relative fluorescence of the dye was proportional to pH. Kinetic rate constants (k) were compared as different values of k correspond to different ghost permeabilities [28]. From the deduced alkalinisation rate constants (k), the apparent NH_3_ permeability was calculated following the equation P’_NH3_=k_exp_.(V_0_/ SA), with V_0_/ SA the ghost-volume/surface-area ratio. Unitary permeability was calculated as Punit NH_3_ = P'_NH3_ hRhAG x1/ N/SA with N/SA the number of sites per surface unit. Ghost diameter measurements were performed using an Axio Observer Z1 microscope (equipped with an AxioCam MRm camera) as described [29].

## Results

### hRhAG, RhD and Rhc membrane protein expression in MEL-C88 erythroleukemia cell line

Expression of human hRhAG, RhD and Rhc proteins in a murine context was assessed using cDNA constructs under control of the EF1α promoter. In MEL-C88, hRhAG protein could be expressed alone as seen by flow cytometry, and stable cell lines established (Figure 1A, left). With the β-globin LCR/promoter/enhancer (Figure 1A, right), hRhAG was expressed, at a lower level. This model also demonstrated that human RhD and Rhc antigens reached the cell membrane only when co-expressed with hRhAG (Figure 1B). In both cases erythroid differentiation by DMSO treatment increased expression in transient transfection. Human RhD expression was dose-dependent on hRhAG expression in MEL-C88 co-transfected by *hRHAG* and *RHD* (Figure 1C).

These experiments showed that in presence of hRhAG, human RhD and Rhc antigens could be expressed in a murine context, whereas MEL-C88 cells transfected with the human *RHD* transgene alone do not express the RhD protein.

### RHD transcript but no protein expression in mice transgenic for RHD_ BAC1

Transgenesis was carried out using *hRHAG* and *RHD* genomic constructs from a human BAC library. No transgenic founder could be obtained for *hRHAG*; one was identified for RHD (TG_*RHD*_BAC1) (Figure S1B, Southern blot) and crossed with a C57Black6/SJL male. Erythrocytes of transgene-positive F1 mice did not react with a panel of anti-RhD monoclonal antibodies recognizing different epitopes or a polyclonal anti-RhD (data not shown). Crossing F1 hemizygotes yielded 6/9 pups carrying the *RHD* transgene, but none expressed RhD, as shown by flow cytometry 3). Real-time RT-PCR on F2 blood samples yielded specific amplification, indicating presence of *RHD* mRNA, and cloning and sequencing of the RHD cDNA from bone marrow showed a correct RhD coding sequence (not shown). 

As with MEL-C88 data, these results strongly suggested that human RhD protein would not be expressed in mouse erythroid cells in the absence of human RhAG despite the presence of murine Rhag. 

### Expression of hRhAG, but not hRh alone, in transgenic mice derived with pGSEL1 vectors

**Figure 2:**
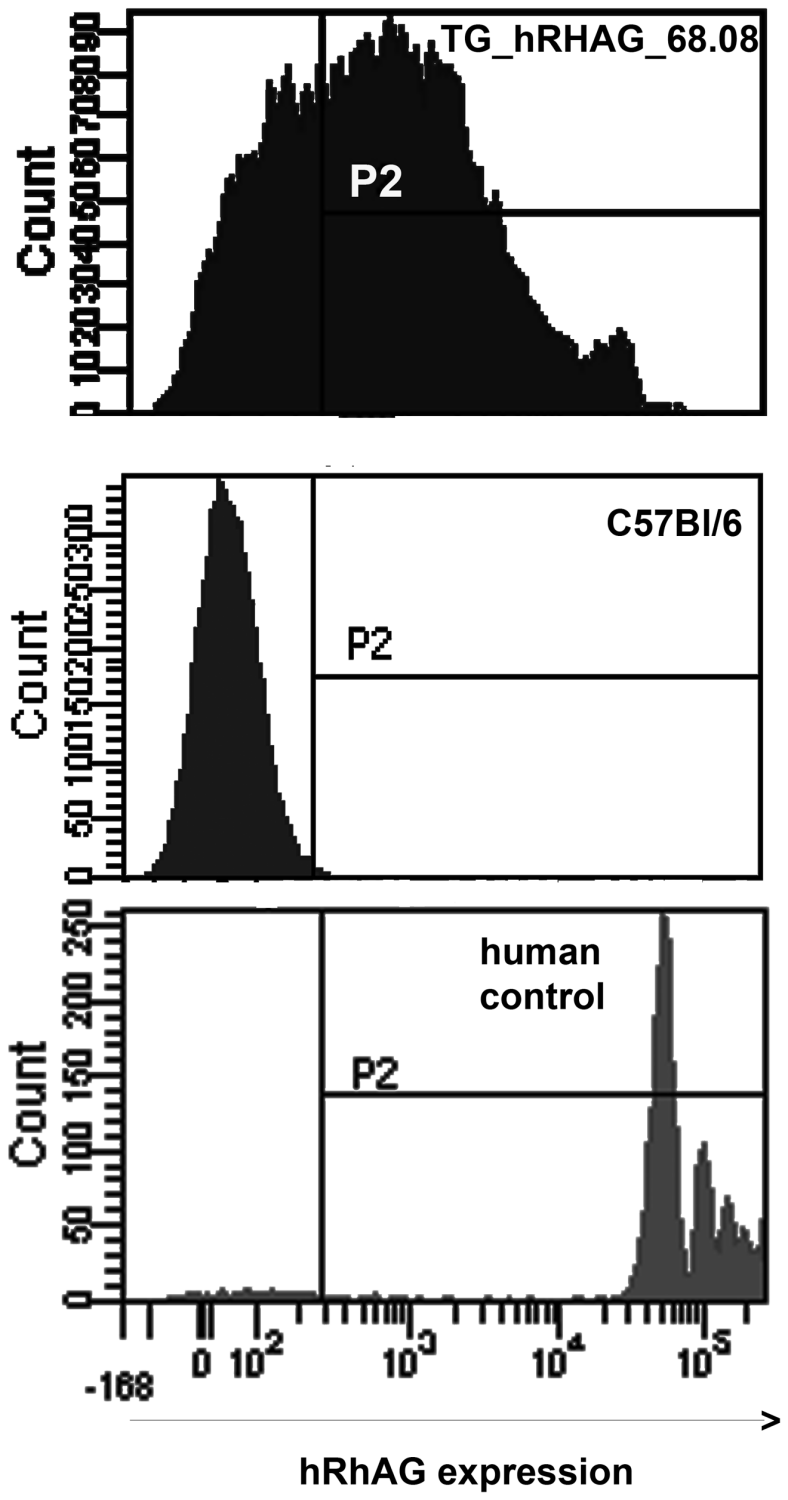
Expression of hRhAG in red blood cells of transgenic mouse line. The *hRHAG* transgene is under control of the erythroid-specific β-globin promoter and LCR (see Figure 1). Patterns of flow cytometry (P2= positive threshold) with anti-hRhAG (LA18.18) for TG_*hRHAG*_68.08 (top), C57Bl/6 control (middle) and a human RBC sample (bottom) shown for comparison.

**Figure 3:**
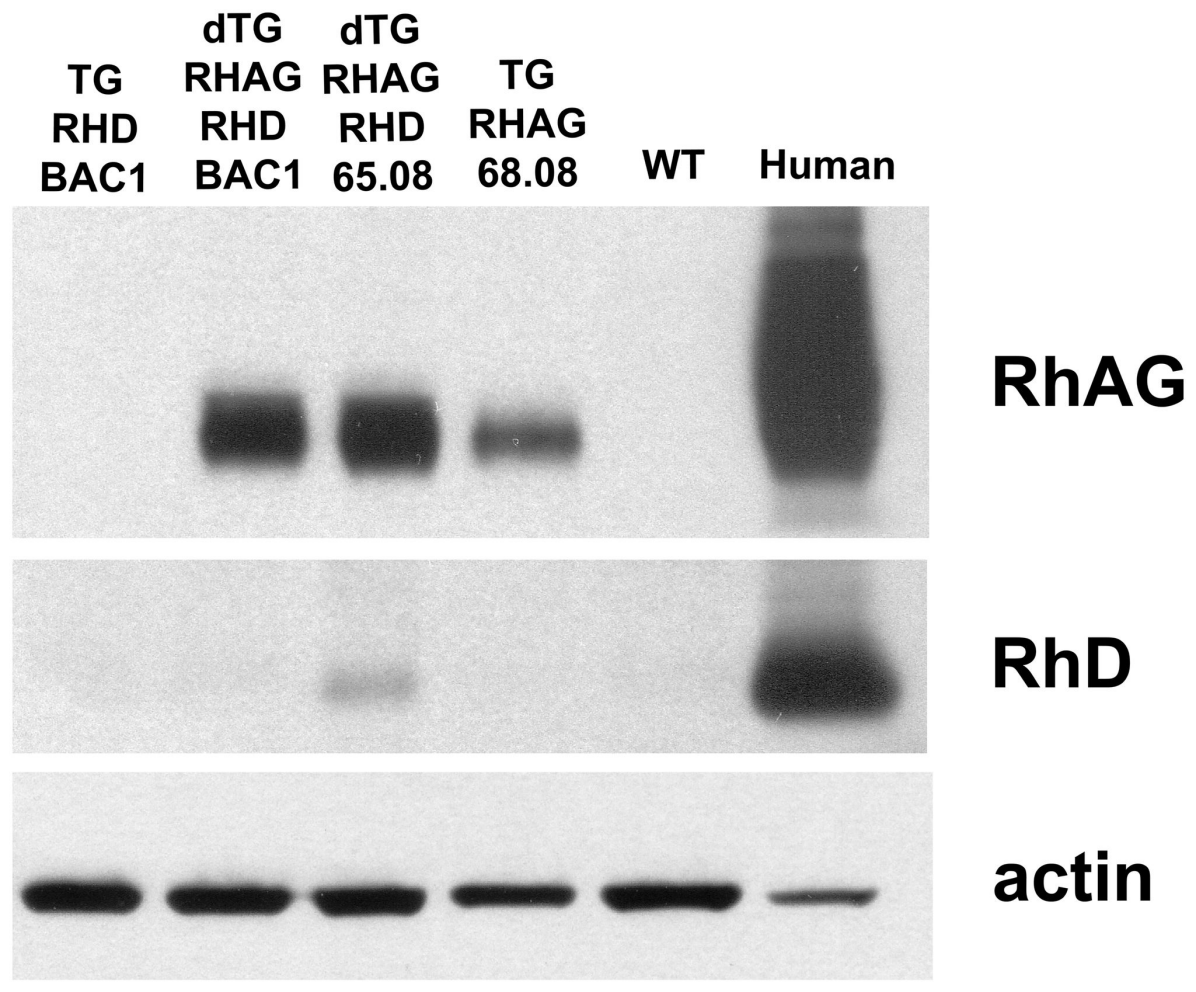
Immunoblot analysis of hRhAG and RhD expression in transgenic mice. Immunoblot from red cell ghost preparations immunostained with anti-hRhAG (LA18.18), anti-RhD (LOR15C9), and anti-actin as loading control. Lane 1= *RHD* single transgenic TG_*RHD*-BAC1, lane 2 = double transgenic cross of TG_*hRHAG*_68.08 with RHD-BAC1, lane 3 = double transgenic cross of TG_*hRHAG*_68.08 with TG_ *RHD*_65.08, lane 4 *hRHAG* single transgenic TG_*hRHAG*_68.08, lane 5=WT and lane 6= human RhD-positive control. hRhAG protein is seen in all mice with the *hRHAG* transgene, whereas RhD expression is detected only in the double transgenic mouse carrying both human *RHAG* and *RHD*_65.08 transgenes.

Since BAC injection did not produce mice transgenic for *hRhAG*, nor mice expressing RhD on red cells, transgenesis of *hRHAG* and *RHD* cDNA under control of the β-globin LCR/promoter/enhancer was carried out using C57Bl/6 and B6/CBA mice. No founders were obtained on C57Bl/6 background. A transgenic line for *hRHAG*, TG_*hRHAG*_68.08, was obtained with B6/CBA (Figure S1A, Southern blot). These mice were positive for erythrocyte membrane expression of hRhAG as confirmed by flow cytometry (Figure 2), with 45 to 75% positive erythrocytes in individual mice, and hRhAG sites about ten times lower than on human erythrocytes (i.e. 8000 as compared to 80 000 sites) (not shown). Human RhAG was evidenced by immunoblot on TG_*hRHAG*_68.08 erythrocyte membranes, not on TG_*RHD*_BAC1, or WT controls (Figure 3). Glycosylation of hRhAG appeared more limited in mouse than human erythrocytes, where a large diffuse band from 30 to 70 kDa can be detected (Figure 3). 

A transgenic line, TG_*RHD*_65.08, was also obtained for *RHD* on B6/CBA background (Figure S1A). Again, no RhD was detected on the erythrocytes (Figure S2), indicating that co-expression of *RHD* and *hRHAG* transgenes is required.

### Mice co-expressing hRhAG with RhD under autologous or β-globin promoter

Transgenics TG_*RHD*_BAC1 and TG_*RHD*_65.08 were crossed with TG_*hRHAG*_68.08, and erythrocytes from the double transgenic mice (dTG_*RHD*_BAC1 and dTG_*RHD*_65.08, respectively) were analysed for expression of hRhAG and RhD. By immunoblot RBCs from these double transgenic mice both expressed hRhAG, but only dTG_*RHD*_65.08 showed a (low) level of RhD antigen expression (Figure 3).

**Figure 4:**
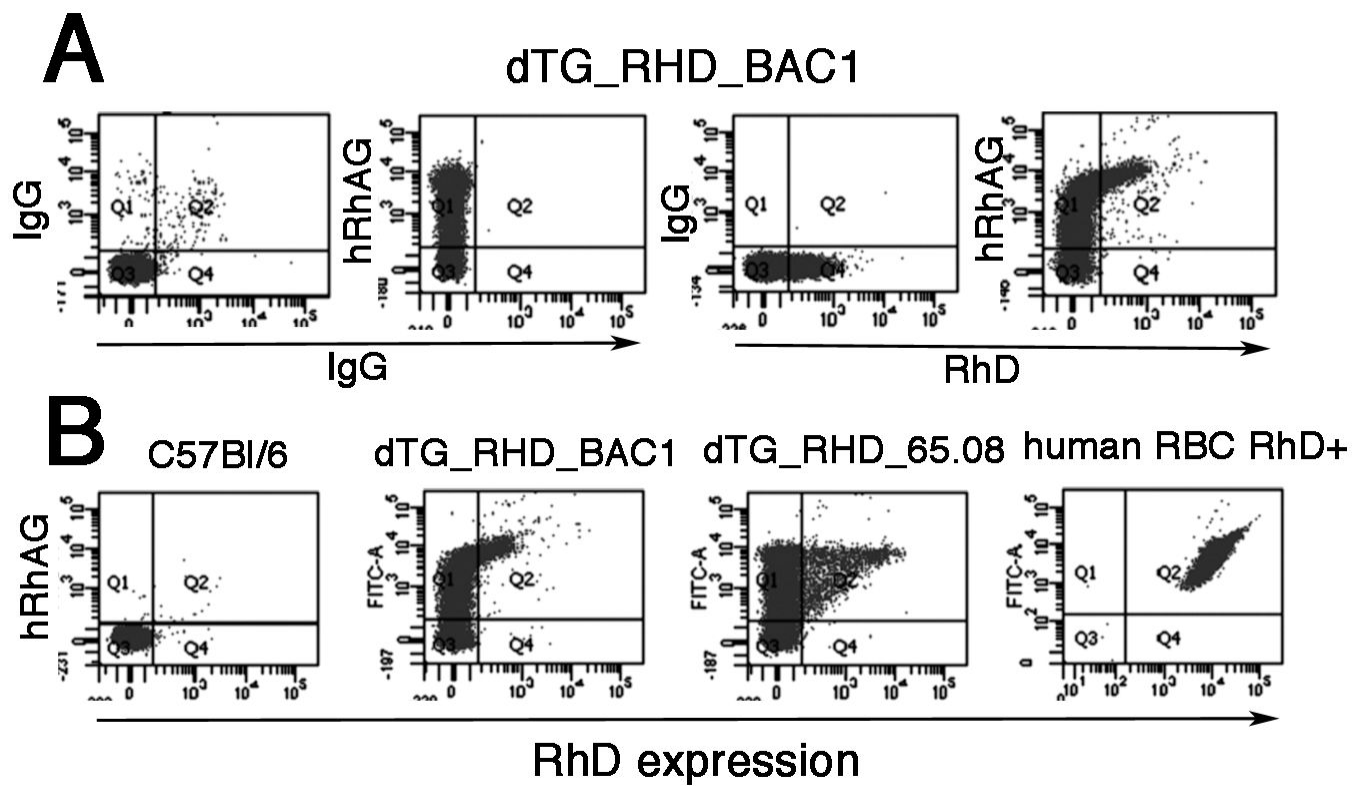
hRhAG and RhD are expressed in erythrocytes of double transgenic mice. Flow cytometry analysis of erythrocytes from double transgenic mice dTG_*RHD*_BAC1 and dTG_*RHD*_65.08 derived from crosses of TG_*hRHAG*_68.08 with *RHD*-BAC1 and TG_ *RHD*_68.08, respectively. (**A**) From left to right: red cells from a dTG_*RHD*_BAC1 mouse labelled for IgG control, hRhAG (LA18.18), RhD (LOR15C9), and both anti-hRhAG and RhD, illustrating compensation set-up. (**B**) From left to right: red cells from C57Bl/6 WT, a second dTG_*RHD*_BAC1, dTG_*RHD*_65.08 and human red cells labelled with anti-hRhAG and RhD.

Flow cytometry (Figure 4) of RBCs from dTG_*RHD*_BAC1 and dTG_*RHD*_65.08 mice showed that both hRhAG and RhD were expressed, with only hRhAG-positive erythrocytes expressing the RhD antigen, a relationship noted in MEL cells (see Figure 1C). Similarly to single transgenic hRhAG mice, 40-70% of RBCs were hRhAG-positive (MFI 10-20% of value for human RBCs). RhD expression levels and the percentage of positive erythrocytes (2 - 15% in individual mice) remained low in both double transgenic lines, as compared to the strong expression on control human erythrocytes (Figure 4B, last dot plot). Interestingly, RhD in dTG_*RHD*_BAC1 was detected only from a high “cut-off” level of hRhAG expression, while in dTG_*RHD*_65.08 (under β-globin regulation), erythrocytes positive for RhD were observed for variable levels of hRhAG, (compare second and third plots Figure 4B). 

Results similar to those with LOR15C9 anti-RhD (epitope Ep3.1) were observed with MAbs H2D5D2F5 (epitope Ep6.2) and LFB R593, (epitope Ep13.1) [30,31], as well as with Rhophylac polyclonal anti-RhD (not shown).

### Expression levels for human *RHD* and mouse *Rh* genes

Expression levels for mouse *Rhd* and human *RHD* were compared in single transgenic TG_RHD_BAC1 or double transgenic dTG_RHD_BAC1 mice, using mouse transferrin receptor1 as endogenous control (Figure S3). Analysis was caried out on the BAC transgenic line as this allowed the use of an exon-spanning probe for *RHD*. We found that when the BAC1_RHD transgene was present, *human RHD* transcript expressed at a level similar to mouse *Rhd* transcript. Though human RhAG protein was necessary for RhD protein membrane expression, presence or absence of a human *RHAG* gene did not change the level of *RHD* transcript.

### Interaction of hRhAG with mRh proteins on erythrocytes of mice transgenic for hRhAG

#### Co-Immunoprecipitation of mRh but not mRhag with hRhAG

**Figure 5:**
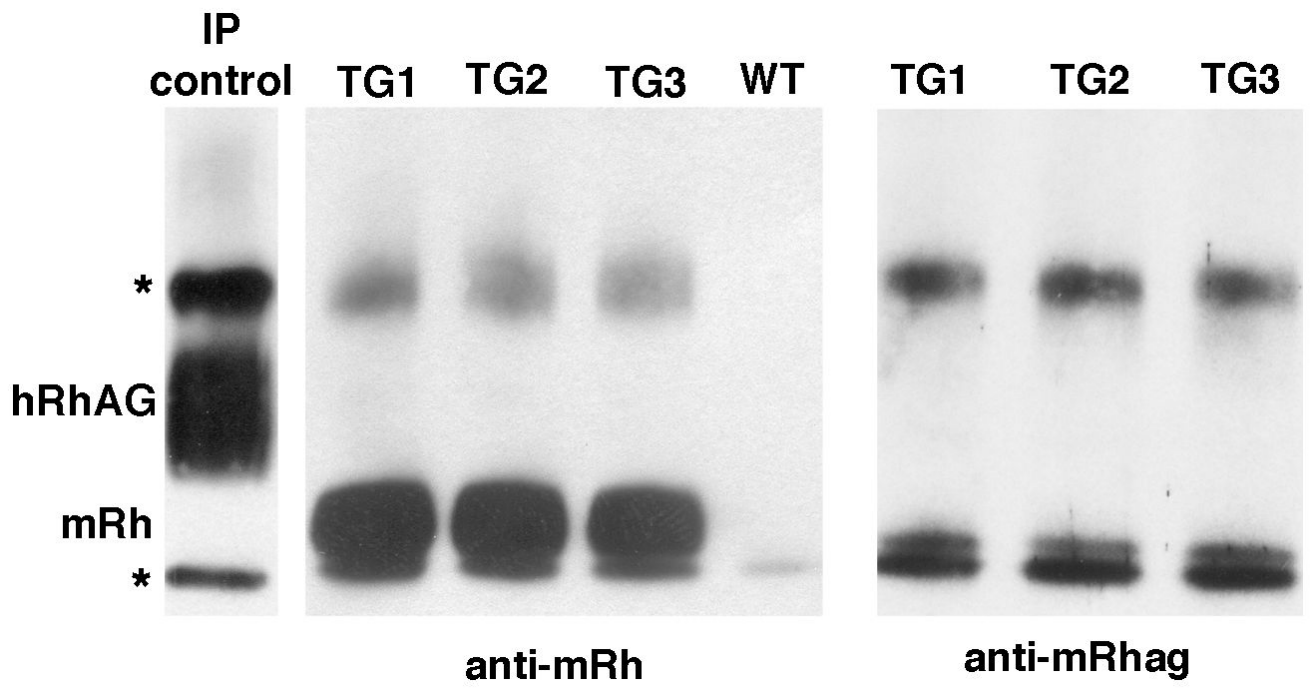
hRhAG co-immunoprecipitates with mRh, not mRhag. Immunoprecipitation (IP) was performed with anti-hRhAG (LA18.18) from erythrocyte ghosts of TG_*hRHAG*_68.08 and WT mice. IP control TG_*hRHAG*_68.08(left), probed with anti-hRhAG, showing that hRhAG was correctly immunoprecipitated from RBC ghosts of TG_*hRHAG*_68.08 transgenic mice. (middle) IP of TG_*hRHAG*_68.08 ghosts (TG1, TG2, TG3) and WT, probed with anti-Rh (MPC8) and (far right) IP of TG_*hRHAG*_68.08 ghosts probed with anti mRhag. Control reactions for the latter result are shown in Figure S4. Immunoglobulin heavy and light chain positions are shown by an asterisk (*).

Immunoprecipitation of hRhAG by LA18.18 was performed on ghosts of TG_*hRHAG*_68.08 erythrocytes, and immunoblots of immunoprecipitated material were probed with polyclonal antibodies reacting with mRh (MPC8), mRhag, or LA18.18 as an immunoprecipitation control. The control showed that hRhAG was correctly immunoprecipitated from red cell ghosts of TG_*hRHAG*_68.08 transgenic mice (IP control on Figure 5,left) and migrated as a diffuse band of the expected size. 

LA18.18 immunoprecipitates from three transgenic TG_*hRHAG*_68.08 mice (TG1, 2 and 3) and a WT littermate were analyzed by SDS-PAGE and immunostained with cross-reactive anti-Rh antibody MPC8. A strong signal at the expected size of Rh protein was detected in the three *hRHAG* transgenic mice, but not in WT (Figure 5, middle). This band represents mRh protein, as there is no human Rh in these mice.

In contrast, when LA18.18 immunoprecipitates from TG_*hRHAG*_68.08 were immunostained with the antibody to mRhag, no signal could be detected (Figure 5, right) indicating that mRhag did not co-precipitate with hRhAG. Controls for this last reaction are shown in Figure S4.

Thus, only mRh proteins are detected in LA18.18 immunoprecipitates, suggesting the presence of mixed-species oligomers of hRhAG and mRh proteins, but not of hRhAG with mRhag.

### Re-expression of mRh on erythrocytes of mRhag knockout mice transgenic for hRhAG

To examine whether mouse Rhag protein competes with human RhAG, expression of hRhAG in the absence of mRhag was investigated by crossing TG_*hRHAG*_65.08 mice with *mRhag* knockout (KO) mice [19]. Flow cytometry of RBCs from siblings of these crosses showed presence of hRhAG, but no increase of expression compared to WT/*hRHAG* mice: in one group of siblings the estimated number of hRhAG sites was 9534+2300 for mRhag^-/-^/hRHAG *(n*=4), 8567+2030 for mRhag^+/-^/hRHAG *(n*=4) and 9129 and 7648 for two *mRhag* ^*+/+*^ */hRHAG*. The number of sites remained approximately ten times less than for human erythrocytes (in this experiment 8x10^4^ sites). 

**Figure 6:**
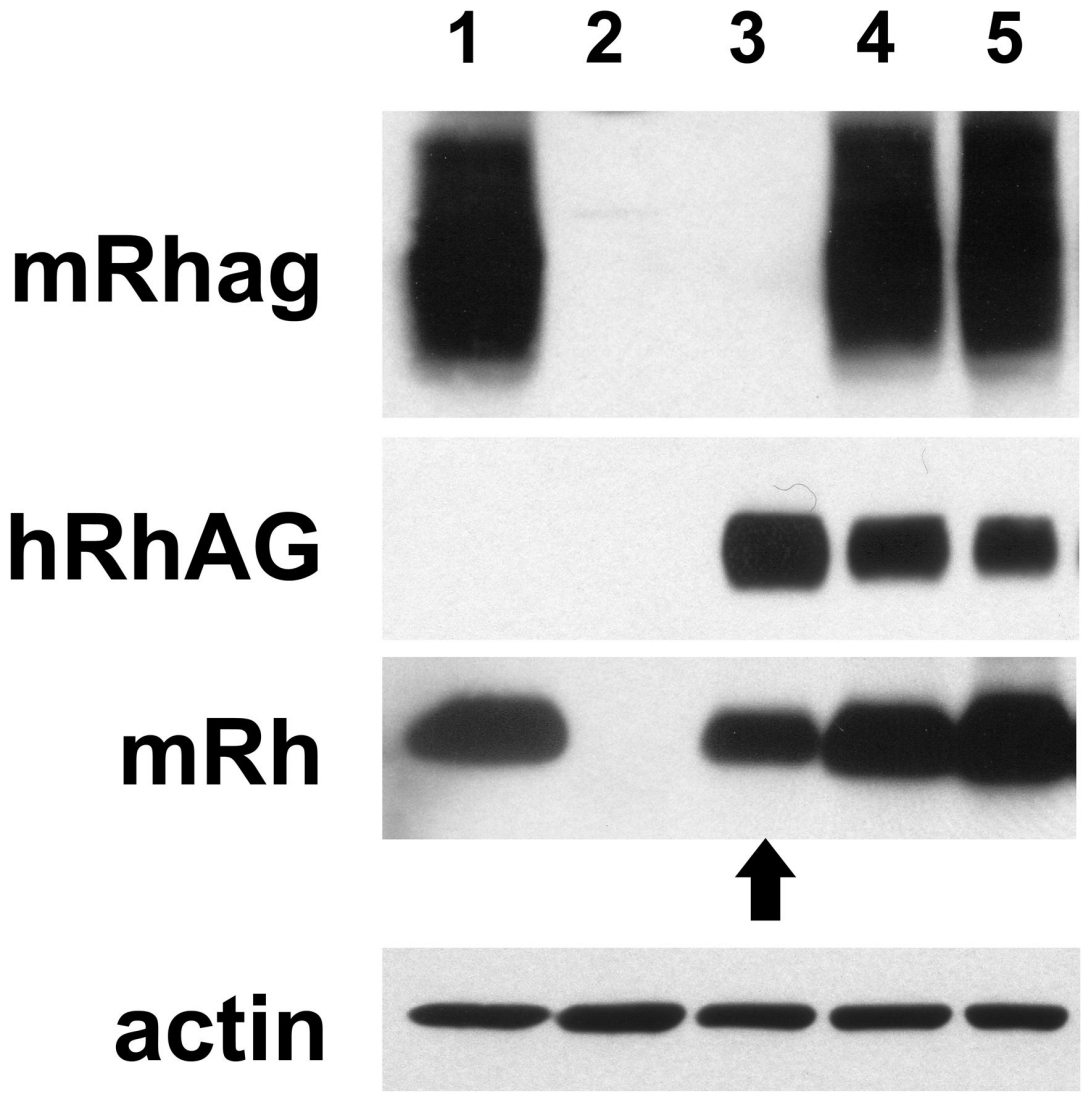
Expression of hRhAG in *mRhag* KO mouse allows re-expression of mRh on the erythrocyte membrane. Immunoblot analysis with anti-mRhag (PAb), anti-hRhAG (LA18.18) and MPC8 (PAb anti-hRh cross-reacting with mRh) of erythrocytes from mice expressing the *hRHAG* transgene on different genetic backgrounds. Lane 1: *mRhag* ^*+/-*^ mouse ; lane 2: mRhag^-/-^ mouse ; lane 3: transgenic *hRHAG* on a *mRhag* ^*-/-*^ background ; lane 4: transgenic *hRHAG* on a *mRhag* ^*+/-*^ background ; lane 5: transgenic *hRHAG* on a wild type background. The arrow shows the re-expression of mRh in a transgenic *hRHAG* mouse on a *mRhag* ^*-/-*^ background (compare lanes 2 and 3).

On immunoblot (Figure 6), ghosts from WT (lane 1) but not *mRhag* ^*-/-*^ animals (lane 2) expressed mRhag and mRh proteins [19]. RBC ghosts from *hRHAG* transgenic mice on *mRhag* KO background (*mRhag* ^*-/-*^ */TG_hRHAG*, lane 3) expressed hRhAG at a slightly higher level than in transgenics on *mRhag* ^*+/-*^ (lane 4) or *mRhag* ^*+/+*^ (lane 5) background, but this remained much lower than that of mRhag in heterozygous or WT mice. Of note, in mouse erythrocytes, hRhAG appears less diffuse than does the endogenous mRhag (and also than hRhAG from human erythrocytes- see Figure 3, above), a consequence of differerences in glycosylation.

Most interestingly, in *mRhag* ^*-/-*^, expression of transgenic hRhAG allowed delivery to the erythrocyte membrane of mRh protein (lane 3), not present on RBCs of *mRhag* ^*-/-*^ mice (lane 2). These findings corroborate the immunoprecipitation experiments, and strongly favour the existence of complexes associating hRhAG and mRh. 

### Ammonium transport in erythrocytes of mRhag^-/-^ mice expressing hRhAG/mRh oligomers

To examine ammonium transport in erythrocytes expressing the heterologous RhAG complexes, pH-dependent fluorescent changes of resealed red cell ghosts from *hRhAG* transgenic *mRhag* KO mice (*mRhag* ^*-/-*^ */hRHAG*) and their *mRhag* KO (*mRhag* ^*-/-*^) controls, subjected to a 10 mEq inwardly directed ammonium gradient in iso-osmotic conditions, were measured with a stopped–flow instrument [28]. The alkalinisation rate constants (k) of ghosts, calculated from intracellular pHi elevation, were 1.45 ± 0.04 s^-1^ for *mRhag* KO and 1.71 ± 0.08 s^-1^ for *hRhAG* transgenic *mRhag* KO animals. The size of ghosts, as measured by light microscopy, was the same for the two genotypes respectively 4.73 ± 0.39 and 4.68 ± 0.42 µm. Thus, *hRhAG* transgenic *mRhag* KO erythrocytes showed a modest but significant (p<0.001) increment in ammonia transport when compared to *mRhag* KO. The apparent unitary permeability to NH_3_ (P_unitNH3_) per hRhAG was 2.0 E-0.3 (Table S2), similar to that measured in normal human erythrocyte ghosts (1.89 E-0.3 [28] and 2.19 E-0.3 calculated from data in [29]), indicating that human hRhAG protein expressed in mouse retains its function, even when a fraction is associated with the mouse mRh protein.

## Discussion

This is the first description of transgenic mouse lines expressing the human RhD protein. RhD could be observed only in mice transgenic for both *RHAG* and *RHD* human genes, consistent with the Rh complex model [7-9,32]. 

Expression of recombinant hRhAG, RhD and Rhc in the murine context was first examined using MEL-C88, a cell line previously used to express human blood group antigens, though not Rh [33]. In MEL cells hRhAG was expressed alone, but human Rh antigen (D and c) expression strictly required hRhAG, as expected from analysis of Rh_null_ variants (reviewed in [8], expression studies using human K562 and HEK293 transfectants [27] and the study of *mRhag* ^*-/-*^ mice [19].

Expression of hRhAG/Rh proteins under control of the human β-globin LCR/ promoter/ enhancer did not significantly improve after erythroid differentiation, when compared to the EF1α promoter. MEL transfectants expressed relatively low levels of hRhAG and still lower levels of RhD or Rhc antigens, contrasting with expression levels ‘similar to those in RBC’ reported for Knops, Kell and Duffy antigens [33]. However, these antigens are expressed on red cells at much lower levels than RhAG/Rh antigens which have a complex membrane topology and whose assembly, traffic and stabilisation in the red cell membrane are currently undefined. Low expression levels had been observed when human K562 erythroleukemia cells, which express substantial amounts of endogenous hRhAG were forced to express human Rh antigens by retroviral- or plasmid-mediated cDNA transfer [27,34], even when co-expressed with recombinant Band 3, reported to enhance Rh antigen expression [35,36]. Of note, upon erythroid differentiation of MEL-C88/hRhAG cells by DMSO, murine band 3 was induced (from an initial 4% to 64% on day 6) and hRhAG expression was also enhanced, consistent with the existence of a macro-complex associating the Rh complex and Band 3 [37]. However, since RhD expression level was directly correlated to hRhAG level in both MEL-C88 (Figure 1C) and K562 cell lines [27], it is postulated that hRhAG represents the essential limiting factor for Rh(D or c) protein expression.

In MEL cells the differentiation process might not have developed normally or gone to completion. In the transgenic mice, analysis of RBCs which had followed the entire murine erythroid differentiation programme *in vivo* was possible. 

As in the cell lines, animals transgenic for the *RHD* gene alone (in the absence of *hRHAG*) did not express any RhD antigen, though transcript for human *RHD* was present, suggesting that endogenous mRhag is unable to assemble and/or transport human RhD protein to the red cell surface. In the *hRHAG* transgenic line, the mean level of hRhAG on RBCs, though highly heterogeneous, was about 10% of that on human erythrocytes. hRhAG could be co-immunoprecipitated with endogenous mRh, but not with mRhag, indicating a potent interaction between hRhAG and mRh. When *hRHAG* transgenic animals were developed on an *mRhag* ^*-/-*^ [19], as compared to a WT background, the level of hRhAG on their erythrocytes was not significantly increased. This suggests that the low levels of hRhAG are not simply a result of competition with mRhag for transport to the membrane, but might reflect differences in Rh complex processing. Very interestingly, immunostaining of RBC membrane proteins from the mRhag^-/-^ mice transgenic for hRhAG revealed that mRh, absent from the mRhag *null* RBC membranes, was re-expressed when hRhAG protein was present. This result was consistent with the formation and trafficking to the membrane of a mixed-species oligomer complex between hRhAG and mRh.

From these cellular expression models and single transgenic mice, it appears that hRhAG-mRh and hRhAG-RhD oligomers are present in the mouse cells in addition to endogenous mRhag-mRh oligomers, but not associations of mRhag-RhD or mRhag-hRhAG. We analyzed the interactions between human and mouse Rh/RhAG proteins in light of 3D structure of human RhCG, solved at 2.1 Å resolution (pdb 3HD6) [12] to build accurate models for the human RhAG/RhD and mouse Rhag/Rh subunits, within the context of a trimeric architecture. These models are refined relative to those we previously published, based on the bacterial ammonia transporter AmtB [38]. Information collected from careful examination of interfaces between RhAG and RhD subunits, within the human RhAG/RhD and mouse Rhag/Rh heterotrimers, is summarized in Figures S5,S6,S7. The main observation deduced from this analysis is that the trimeric interface includes several aromatic amino acids, some of which are well conserved between the subunits and between the human and mouse sequences. Striking differences between the human and mouse sequences concentrate in two symmetrical external areas of the interfaces between the RhAG and Rh subunits (Figures S5,S6,S7). This is certainly not the only factor to be considered, due to the large surface involved in the trimer assembly, but the region highlighted here is likely to be one of those which may play a critical role.

When the mice were transgenic for both *hRHAG* and *RHD* on a WT background, the two human proteins, hRhAG and RhD, were expressed on RBCs, as opposed to the absence of RhD expression in the *RHD* single transgenics. The human RhD polypeptide expressed was recognized by polyclonal anti-D Rhophylac as well as by monoclonal anti-Ds reacting with different epitopes, respectively Ep 3.1 (LOR15C9) Ep6.2 (H2D5D2F5) and Ep13.1(LFB-R593) [30], and thus would appear to be assembled in the membrane in identical fashion to that in human red cells.

When compared to MEL cells, expression of the two proteins was less directly correlated, and hRhAG appeared to be in excess of RhD antigen. This suggests that hRhAG might not be the only limiting factor for RhD antigen expression, even taking into account a possible competition of mRh with RhD. A future analysis of double transgenics on an *mRhag* ^*-/-*^ *mRh* ^*-/-*^ background may clarify this issue. Thus, although hRhAG is essential for RhD expression, it is not sufficient to promote full membrane expression of Rh antigens at the cell surface. 

Factors that may limit heterologous hRhAG/Rh expression in transgenic animals at the transcription level include insertion site and transgene copy number, not studied here, as only one transgenic line could be established in each case. The difference in expression pattern of hRhAG/RhD under BAC or β-globin regulation might be linked to differing nuclear organization of transcription for the promoters (reviewed in Cope et al.[39]). Transgene expression outside of the original gene locus is often anarchic, either very high or very low, and may be prone to variegation [40]. However, when *RHD* transcript expression was studied in the BAC transgenic lines, we found that found that whenever the BAC1_RHD transgene was present, human *RHD* transcript was expressed, and at a level comparable to that of mouse Rh. We also found that presence or absence of a human RHAG gene did not impact the level of *RHD* transcript. These observations are in favor of a post-transcriptional nature of the limitations in RhD expression.

A number of factors may play a role in intracellular traffic and/or cell surface expression of RhD and RhAG on mouse RBCs. 

Impairment of N-glycosylation has been shown to disrupt the targeting and stability of membrane protein expression, notably in the case of cytokine receptors, and transporters (reviews in [41,42]), though conservation of core glycosylation was sufficient for correct cell surface expression of the cystic fibrosis transmembrane conductance regulator [43]. Post-translational processing of human RhAG in mouse and human erythroid cells differs, our results show that hRhAG glycosylation is reduced in the mouse, bands are less diffuse, and always below 50kDa on SDS-PAGE. How altered glycosylation of hRhAG might affect trafficking mechanisms for efficient cell surface expression of Rh antigens, or be a reflection of trafficking defects, is currently not clear. 

Differences in regulation of trafficking of RhAG to the cell membrane in man and mouse have been discussed recently [44], with emphasis on the putative role of protein 4.2 and GPB (glycophorin B), both present in the Rh macrocomplex. In man, in the absence of protein 4.2 there is increased glycosylation of RhAG [20,21], this is also seen in GPB-deficient RBCs [45]. In contrast, in the mouse, endogenous mRhag glycosylation is not affected by protein 4.2 deficiency [20,21]. However, in both cases (man or mouse), expression of Rh antigens is normal in 4.2-deficiency [20,21]. 

The lack, or inadequacy of a mouse protein partner for transit or membrane stabilisation could also affect hRhAG trafficking. In mice the *Gypb* gene is absent [46], but this accessory protein is also dispensable in the human Rh complex, since GPB-deficient individuals express Rh blood group antigens normally [45]. CD47 is independent of the Rh complex in mouse. As concerns ICAM4, erythrocytes of human LWa-/LWb- individuals, lacking ICAM4, are not deficient in Rh antigens [7], and we found no difference in ICAM4 between transgenic and WT mice (data not shown). Differences implicating these known accessory Rh complex proteins should thus not significantly compromise cell surface expression of the exogenous hRhAG/RhD protein core.

Recent studies have shown that the Rh complex is an important interaction site between the lipid bilayer and the spectrin-based membrane skeleton, that regulate the shape, deformability and mechanical properties of RBCs. Data from protein 4.1R-deficient mice led to the proposal that a proportion of mRh, interacting with the 4.1R-based junctional complex [47], was independent of mRhag. However, in our mRhag-/- knockout and hRhAG transgenic mice, we could detect no fraction of independently bound Rh, and mRh was never found at the red cell membrane without either mRhag or hRhAG. These observations tend not to favor an impact of protein 4.1R on RhD expression, all the more so as, in human protein 4.1*null* red cells, the Rh protein was not altered (unpublished data referred to by [17]). The critical role of ankyrin 1 in Rh-membrane skeleton linkage and the relationship with the Band 3 macrocomplex have also been underlined (reviewed in [17,44]). Ankyrin 1 is an essential partner, anchoring RhAG and Rh proteins at the red cell surface. mRhag and mRh are severely reduced on RBCs from ankyrin1-deficient *nb/nb* mice [48], and (Rh) in ENU *Ank1* null mice [49]. One mechanism for deficiencies of mRhag/mRh in *nb/nb* mice appears to be abnormal sorting during erythroblast enucleation [50]. Whether mouse ankyrin 1 can interact with human RhAG is not known. Homology of subdomain 2 of membrane binding domain in mouse and human ankyrin 1 [48] would be in favor of this, as well as the observation of hRhAG at the mouse RBC membrane. 

No clear differences involving identified partners of the Rh complex appear to explain the reduced efficiency of hRhAG and RhD expression in the mouse RBC – as yet unknown elements in Rh complex trafficking and stability may be implicated.

Our studies demonstrate that it is possible to express the human RhD antigen on erythrocytes in mice, provided the human Rh glycoprotein (hRhAG) is co-expressed, as mouse Rhag appears unable to direct RhD protein to the cell membrane. The development of mice expressing human RhAG and RhD represents a novel approach to dissect Rh/RhAG complex formation, in complement to studies of human erythroid differentiation *in vitro* [51]. The production of transgenic animals for human Rh antigens is a step towards the development of humanized Rh-positive and Rh-negative mouse lines. These should prove useful in modelling the immune response to Rh antigens - allowing analysis of Rh immunisation and mechanisms of antibody-mediated immune suppression in mice whose immune repertoire has developed in the presence of the human Rh proteins. This model could provide a basis for the evalution of the protective mechanisms of anti-Rh D prophylaxis, and an experimental basis for the evaluation of monoclonal antibody efficacy.

## Supporting Information

Table S1 **CEPH RHD and RHAG BACs selected for transgenesis.**
(PDF)

Table S2 **NH3 permeability of mRhag-/- and mRhag-/-/hRhAG+ erythrocyte ghosts.** N= number of RhAG antigen sites estimated as antibody binding capacity (see Methods) ; SA = surface area ; V= volume ; k=.
alkalinisation rate constant ; *background level ; § p< 0.001.
1. Ripoche et al. Proc Natl Acad Sci U S A. 2004;101:17222-17227.
2. Genetet et al. Am J Physiol Cell Physiol. 2012;302:C419-428.
(PDF)

Figure S1 **Transgene structure and Southern blots.** (**A**) *Left*: pGSEL1 vector β-globin-based transgene structure for *hRHAG*, *RHD* and *RHce*. *Right*: Southern blot hybridisation derived from agarose gel-fractionated *Eco*RV-digested genomic DNA of TG_*hRHAG* 68.08 founder (F0) and F1 animals (top) or *Bgl*II-digested genomic DNA of TG_*RHD* 65.08 F0, F1 and F2 (bottom) with WT controls, probed with an 800 bp fragment (5’probes) spanning the 3’end of the β-globin promoter and the 5’end of *hRHAG* cDNA or 5’end RHD cDNA respectively. Lane 1 size marker (**B**) *Left*: RHD_BAC1 contains the *RHD* gene but neither *TMEM50* nor *RHCE* (ub & db: upstream and downstream Rh boxes). *Right*: Southern blot hybridisation derived from agarose gel-fractionated *Eco*RI-digested genomic DNA of RHD_BAC1 F0, F3 and wild type (WT) obtained with an *RHD* exon 4 probe. Lane 1 BAC1 as size reference.
(TIF)

Figure S2 **RhD antigen expression of single and double transgenic mice.** Flow cytometry analysis shows that erythrocytes from *hRHAG-RHD* double transgenic mice (dTG_RHD_BAC1 or dTG_RHD_65.08) obtained by crossing TG_RHD_BAC1 or TG_RHD_65.08 with TG_RHAG_68.08, respectively, express RhD antigen, while erythrocytes from *RHD* single transgenics (TG_BAC1_RHD or TG 65.08_RHD) do not. Red cells labeled for IgG (control) and RhD expression (anti-D LOR15C9).
(PDF)

Figure S3 **mRh or hRHD expression normalized to transferrin receptor expression in RHD and in **RHD**/**RHAG** transgenic mice.** Transcript expression was measured in single (BAC1_RHD) transgenic mice and in mice transgenic for human RHAG and RHD (dTG BAC1_RHD)- four mice of each type. In BAC1_RHD transgenic mice, human RhD transcript was expressed at a level comparable to that of mouse Rh. In double transgenic mice, the presence of the human *RHAG* gene did not increase the level of RHD transcript as compared to the single transgenics (Student’s unpaired t-test p=0.45).
(TIF)

Figure S4 **hRhAG does not co-immunoprecipitate with mRhag: controls.** (*Left*) No signal is detected when LA18.18 immunoprecipitates from TG_*hRHAG*_68.08 and WT ghosts are immunostained with anti-mRhag (*top*). Probing with anti-hRhAG confirms hRhAG immunoprecipitation in the transgenic but not the WT (*bottom*). Positive controls (*middle*): direct immunostaining of red cell ghosts from TG_*hRHAG*_68.08 and WT with the same antibodies showing the presence of mRhag in both samples but of hRhAG in the transgenic line only. Negative controls (*right*) for the immunoprecipitation reaction showing the absence of detectable mRhag or hRhAG signals: LA18.18 + protein G only (lane 1); protein G, no MAb, + TG ghost (lane 2), protein G, no MAb, + WT ghost (lane 3). Samples, run on the same gel, were cut and separated for clarity of presentation. The asterisk (*) indicates the position of immunoglobulin H and L chains.
(TIF)

Figure S5 **Sequence alignments.** Alignment of the sequence of the human RhCG subunit (whose 3D structure has been solved, pdb 3HD6) [12] with the sequences of hRhAG, mRhag and RhD. The human RhCG sequence shares 51 % and 32 % with the hRhAG and RhD sequences, respectively (49 % and 32 % with the mRhag and mRh sequences). The observed secondary structures are shown above the alignment. Squares indicate positions participating in the interface between the subunits of a trimeric assembly, as deduced from the observed significant differences in solvent accessibility between the monomeric and trimeric subunits. Green squares indicate amino acids that are identical between hRhAG and RhD and between the mouse and human sequences, orange those which are different between hRhAG and RhD but identical between the human and mouse sequences, red those which differ between hRhAG and RhD and between the human and mouse sequences.
(PDF)

Figure S6 **Analysis of 3D structures.**Model of the 3D structure of the human RhAG/RhD(2) heterotrimer, based on the experimental 3D structure of the human RhCG homotrimer (pdb 3HD6) [12] and the alignment given in Figure S4. The amino acids participating in the heterotrimer interface are shown and colored according to Figure S4). These involve residues from M0 in one subunit and residues from M7 and M9 in the other subunit. In particular, three amino acids from M7 (F245 in human RhAG, Y243 in human RhD) and M9 (H292, P293 and F294 in human RhAG, S290, P291 and W292 in human RhD) seem to play a critical role in the interface formed with helix M0 (involving human RhD F28 and Y29 and human RhAG F20 and G21). The large substitutions observed with other amino acids in the equivalent mouse sequences in the area of the human RhD M0/RhAG M7-M9 interface (human RhAG F245 with M255, human RhAG H292 with P302, F294 with Y304, human RhD Y29 with C29, H33 with P33 and Y34 with H34) may lead to substantial differences, which may preclude the formation of a stable heterotrimer mRhag/RhD(2) chimera.
(PDF)

Figure S7 **Differences between human and mouse sequences.** Magnification of two areas from Figure S5 concentrating major differences between human and mouse sequences.
(PDF)
