# Supplementary material for: Mice Expressing RHAG and RHD Human Blood Group Genes
Source: PLoS One. 2013 Nov 18;8(11):e80460. doi: 10.1371/journal.pone.0080460 (PMC3832391; doi:10.1371/journal.pone.0080460)
Supplement: Table S1 — CEPH RHD and RHAG BACs selected for transgenesis. (PDF) [file pone.0080460.s001.pdf]

	***RHD*-BAC1**	***RHAG*-BAC18**
BAC name	H0193H09	H0696H07
Library	HindIII	HindIII
Size Kb	250	145
Chromosome assignment	1p	6p
Cytogenetic localisation(s)	1p36.2-1p34	6p21-qter
Method	STS	STS
Date of experiment	2001-01-15	2001-01-15
Center	CEPH	CEPH

Table S1. CEPH *RHD* and *RHAG* BACs selected for transgenesis.
