# Supplementary material for: Mice Expressing RHAG and RHD Human Blood Group Genes
Source: PLoS One. 2013 Nov 18;8(11):e80460. doi: 10.1371/journal.pone.0080460 (PMC3832391; doi:10.1371/journal.pone.0080460)
Supplement: Table S2 — NH3 permeability of mRhag-/- and mRhag-/-/hRhAG+ erythrocyte ghosts. N= number of RhAG antigen sites estimated as antibody binding capacity (see Methods) ; SA = surface area ; V= volume ; k=. alkalinisation rate constant ; *background level ; § p< 0.001. 1. Ripoche et al. Proc Natl Acad Sci U S A. 2004;101:17222-17227. 2. Genetet et al. Am J Physiol Cell Physiol. 2012;302:C419-428. (PDF) [file pone.0080460.s002.pdf]

Mice	RBC ghosts					NH_3 permeability				
	**N**	**Diameter** (µm)	**SA** (μm^2)	**N/SA** (μm^{-2})	**V/SA** (µm)	**T** (°C)	**k** (s^{-1})	**Total** P'_{NH3} ($\mu m.s^{-1}$)	**hRhAG** P'_{NH3} ($\mu m.s^{-1}$)	$P_{unit\ NH3}$ ($\mu m^3.s^{-1}$)
KO *Rhag/ hRHAG*	6746	4.68	68.77	98.1	0.78	15	1.72§	1.3416	0.1961	2.0 E-0.3
KO *Rhag*	600*	4.73	70.25	8.5	0.79	15	1.45	1.1455	0	
RhAG$^{+/+}$ control[1]	81 000	5.0	77.0	1052.3	0.83	15	4.95	4.08	1.99	1.89 E-0.3
RhAG$^{+/+}$ control[2]	81 000	5.8	106	763.8	0.97	15	2.82	2.73	1.67	2.19E-0.3

Table S2. NH_3 permeability of *mRhag*$^{-/-}$ and *mRhag*$^{-/-}$/*hRhAG*+ erythrocyte ghosts
N= number of RhAG antigen sites estimated as antibody binding capacity (see Methods) ; SA = surface area ; V= volume ; k= alkalinisation rate constant ; *background level ; § $p < 0.001$.

1. Ripoche et al. Proc Natl Acad Sci U S A. 2004;101:17222-17227.
2. Genetet et al. Am J Physiol Cell Physiol. 2012;302:C419-428.
