# Supplementary figures and images for: Mice Expressing RHAG and RHD Human Blood Group Genes
Source: PLoS One. 2013 Nov 18;8(11):e80460. doi: 10.1371/journal.pone.0080460 (PMC3832391; doi:10.1371/journal.pone.0080460)

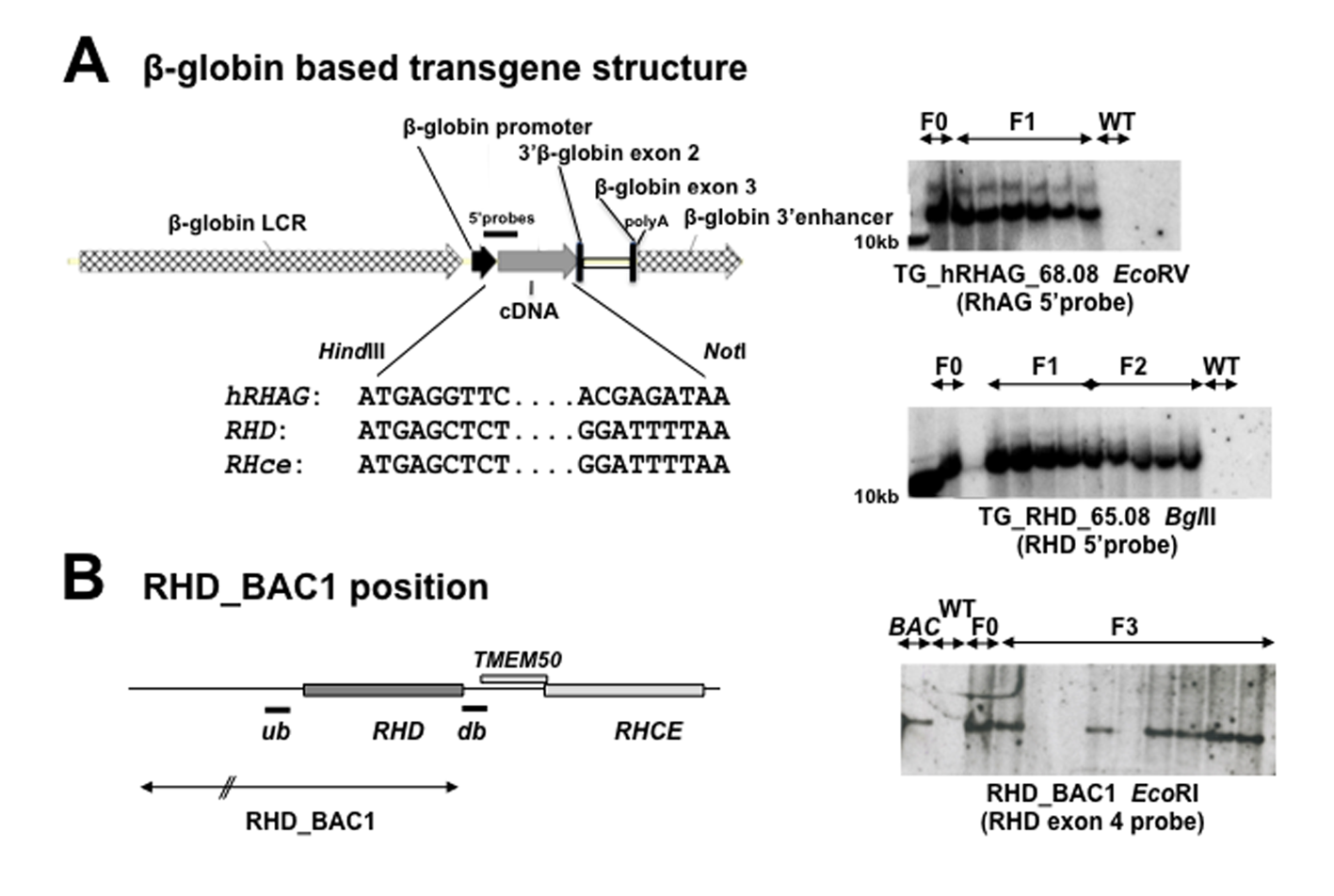

Supplement: Figure S1 — Transgene structure and Southern blots. (A) Left: pGSEL1 vector β-globin-based transgene structure for hRHAG, RHD and RHce. Right: Southern blot hybridisation derived from agarose gel-fractionated EcoRV-digested genomic DNA of TG_hRHAG 68.08 founder (F0) and F1 animals (top) or BglII-digested genomic DNA of TG_RHD 65.08 F0, F1 and F2 (bottom) with WT controls, probed with an 800 bp fragment (5’probes) spanning the 3’end of the β-globin promoter and the 5’end of hRHAG cDNA or 5’end RHD cDNA respectively. Lane 1 size marker (B) Left: RHD_BAC1 contains the RHD gene but neither TMEM50 nor RHCE (ub & db: upstream and downstream Rh boxes). Right: Southern blot hybridisation derived from agarose gel-fractionated EcoRI-digested genomic DNA of RHD_BAC1 F0, F3 and wild type (WT) obtained with an RHD exon 4 probe. Lane 1 BAC1 as size reference. (TIF) [file pone.0080460.s003.tif]

TG\_RHD\_BAC1

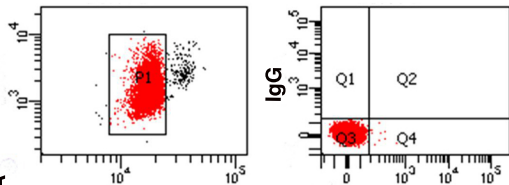

TG\_RHD\_65.08

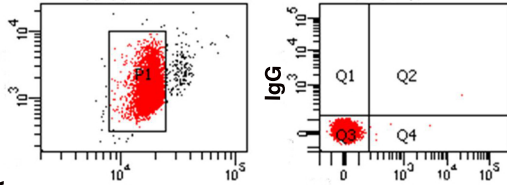

dTG\_RHD\_BAC1

SSC-A

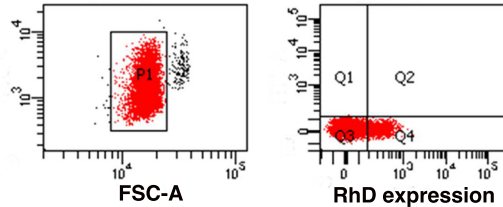

dTG\_RHD\_65.08

SSC-A

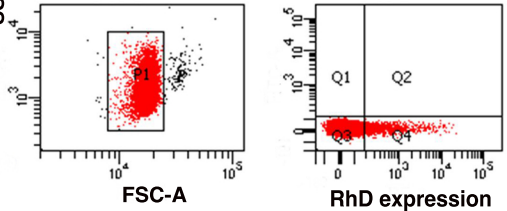

Supplement: Figure S2 — RhD antigen expression of single and double transgenic mice. Flow cytometry analysis shows that erythrocytes from hRHAG-RHD double transgenic mice (dTG_RHD_BAC1 or dTG_RHD_65.08) obtained by crossing TG_RHD_BAC1 or TG_RHD_65.08 with TG_RHAG_68.08, respectively, express RhD antigen, while erythrocytes from RHD single transgenics (TG_BAC1_RHD or TG 65.08_RHD) do not. Red cells labeled for IgG (control) and RhD expression (anti-D LOR15C9). (PDF) [file pone.0080460.s004.pdf]

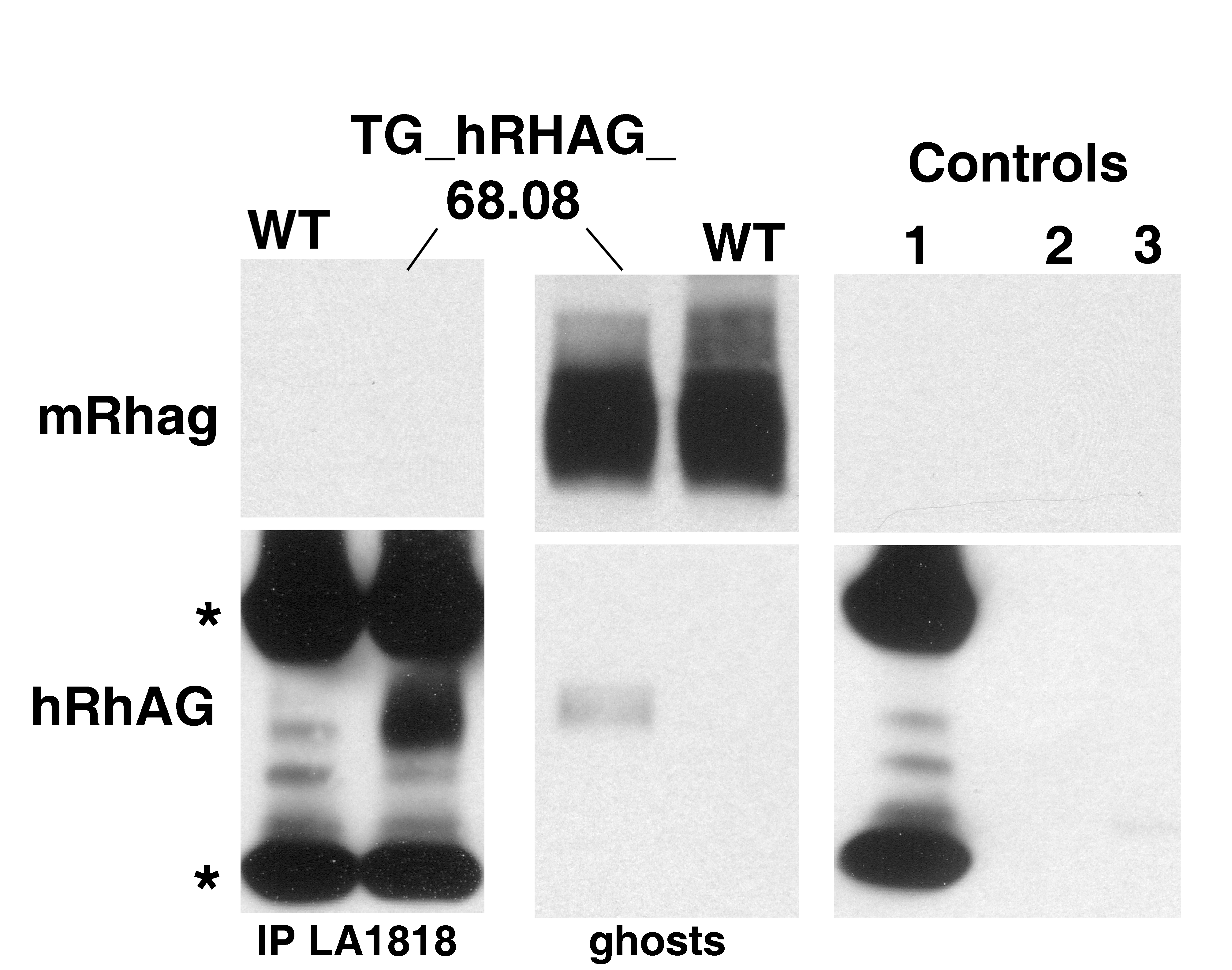

Supplement: Figure S4 — hRhAG does not co-immunoprecipitate with mRhag: controls. (Left) No signal is detected when LA18.18 immunoprecipitates from TG_hRHAG_68.08 and WT ghosts are immunostained with anti-mRhag (top). Probing with anti-hRhAG confirms hRhAG immunoprecipitation in the transgenic but not the WT (bottom). Positive controls (middle): direct immunostaining of red cell ghosts from TG_hRHAG_68.08 and WT with the same antibodies showing the presence of mRhag in both samples but of hRhAG in the transgenic line only. Negative controls (right) for the immunoprecipitation reaction showing the absence of detectable mRhag or hRhAG signals: LA18.18 + protein G only (lane 1); protein G, no MAb, + TG ghost (lane 2), protein G, no MAb, + WT ghost (lane 3). Samples, run on the same gel, were cut and separated for clarity of presentation. The asterisk (*) indicates the position of immunoglobulin H and L chains. (TIF) [file pone.0080460.s006.tif]

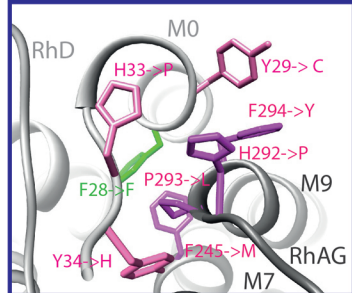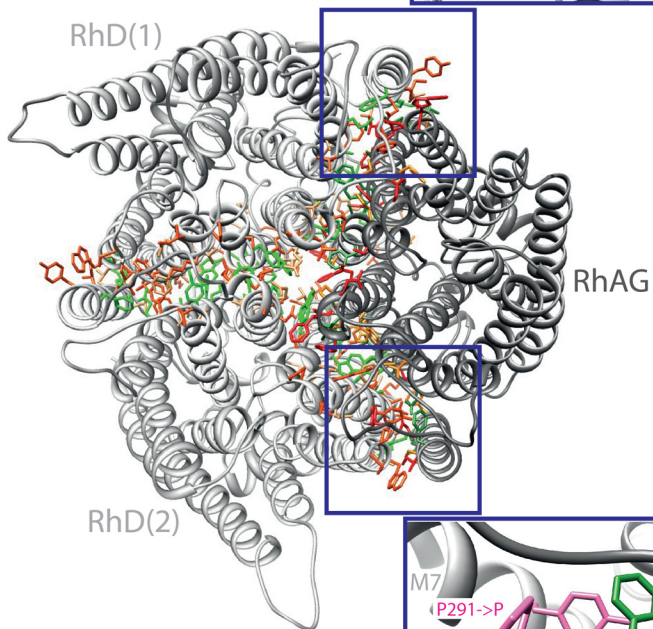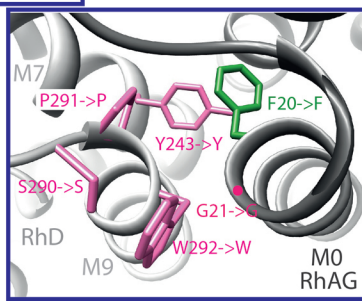

Supplement: Figure S6 — Analysis of 3D structures.Model of the 3D structure of the human RhAG/RhD(2) heterotrimer, based on the experimental 3D structure of the human RhCG homotrimer (pdb 3HD6) [12] and the alignment given in Figure S4. The amino acids participating in the heterotrimer interface are shown and colored according to Figure S4). These involve residues from M0 in one subunit and residues from M7 and M9 in the other subunit. In particular, three amino acids from M7 (F245 in human RhAG, Y243 in human RhD) and M9 (H292, P293 and F294 in human RhAG, S290, P291 and W292 in human RhD) seem to play a critical role in the interface formed with helix M0 (involving human RhD F28 and Y29 and human RhAG F20 and G21). The large substitutions observed with other amino acids in the equivalent mouse sequences in the area of the human RhD M0/RhAG M7-M9 interface (human RhAG F245 with M255, human RhAG H292 with P302, F294 with Y304, human RhD Y29 with C29, H33 with P33 and Y34 with H34) may lead to substantial differences, which may preclude the formation of a stable heterotrimer mRhag/RhD(2) chimera. (PDF) [file pone.0080460.s008.pdf]

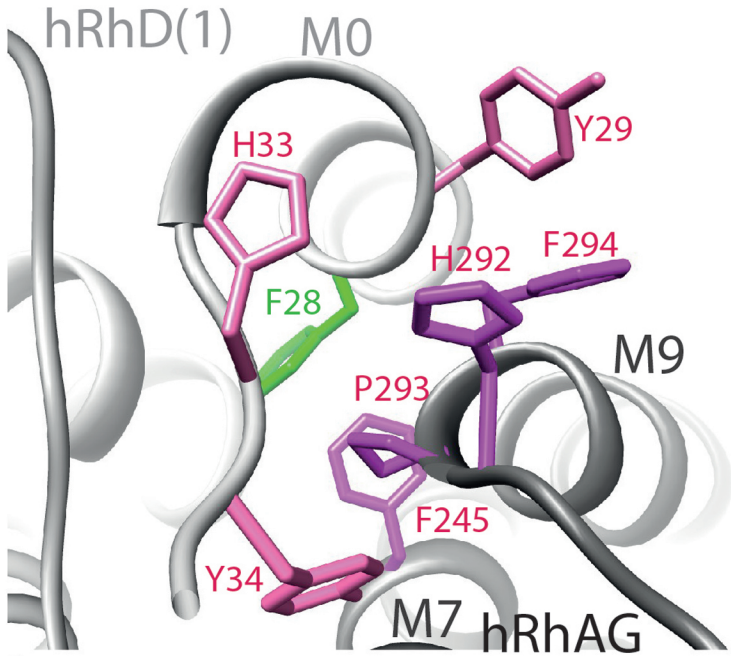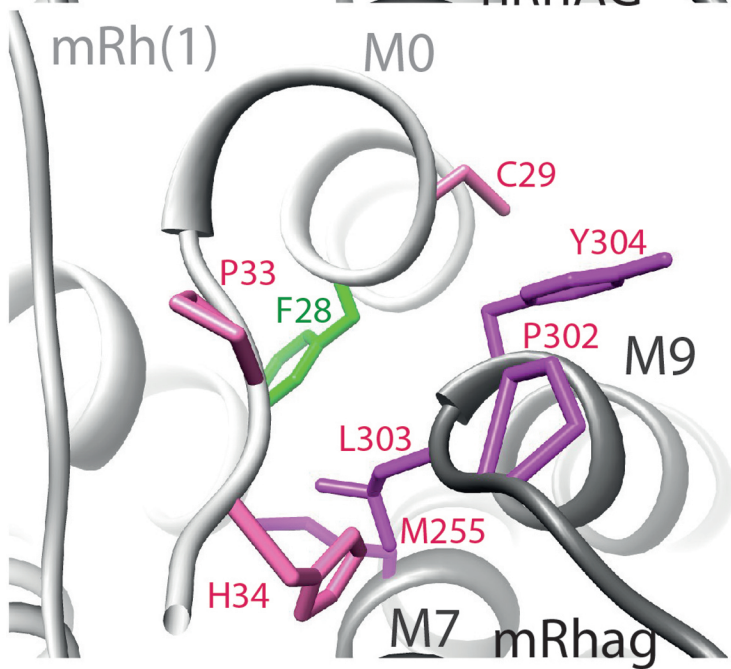

Supplement: Figure S7 — Differences between human and mouse sequences. Magnification of two areas from Figure S5 concentrating major differences between human and mouse sequences. (PDF) [file pone.0080460.s009.pdf]
