# Supplementary material for: Mice Expressing RHAG and RHD Human Blood Group Genes
Source: PLoS One. 2013 Nov 18;8(11):e80460. doi: 10.1371/journal.pone.0080460 (PMC3832391; doi:10.1371/journal.pone.0080460)
Supplement: Figure S5 — Sequence alignments. Alignment of the sequence of the human RhCG subunit (whose 3D structure has been solved, pdb 3HD6) [12] with the sequences of hRhAG, mRhag and RhD. The human RhCG sequence shares 51 % and 32 % with the hRhAG and RhD sequences, respectively (49 % and 32 % with the mRhag and mRh sequences). The observed secondary structures are shown above the alignment. Squares indicate positions participating in the interface between the subunits of a trimeric assembly, as deduced from the observed significant differences in solvent accessibility between the monomeric and trimeric subunits. Green squares indicate amino acids that are identical between hRhAG and RhD and between the mouse and human sequences, orange those which are different between hRhAG and RhD but identical between the human and mouse sequences, red those which differ between hRhAG and RhD and between the human and mouse sequences. (PDF) [file pone.0080460.s007.pdf]

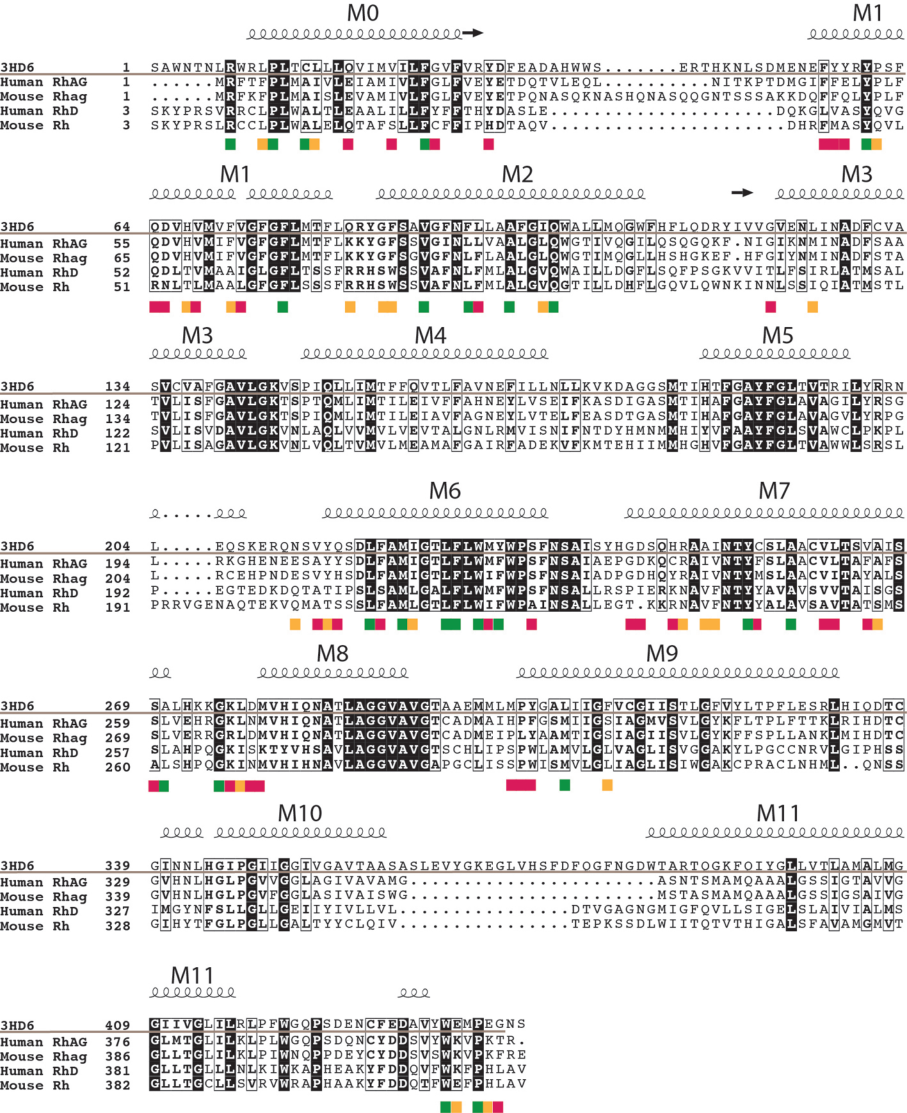
M0
M1
3HD6 1 SAWNTNLRWRLPLTCLLLQVIMVILFGVFVRYDFEADAHWWS.......ERTHKNLSDMENEFYYRYPSF
Human RhAG 1 ......MRFTFPLMAIVLEIAMIVLFGLFVEYETDQTVLEQL..........NITKPTDMGIFFELYPLF
Mouse Rhag 1 ......MRFKFPLMAISLEVAMIVLFGLFVEYETPQNASQKNASHQNASQQGNTSSSAKKDQFFQLYPLF
Human RhD 3 SKYPRSVRRCLPLWALTLEAALILLFYFFTHYDASLE.....................DQKGLVASYQVG
Mouse Rh 3 SKYPRSLRCCLPLWALELQTAFSLLFCFFIPHDTAQV.....................DHRFMASYQVL
M1
M2
M3
3HD6 64 QDVHVMVFVGFGFLMTFLQRYGFSAVGFNFLLAAFGIQWALLMQGWFHFLQDRYIVVGVENLINADFCVA
Human RhAG 55 QDVHVMIFVGFGFLMTFLKKYGFSSVGINLLVAALGLQWGTIVQGILQSQGQKF.NIGIKNMINADFSAA
Mouse Rhag 65 QDVHVMIFVGFGFLMTFLKKYGFSGVGFNLFLAALGLQWGTIMQGLLHSHGKEF.HFGIYNMINADFSTA
Human RhD 52 QDLTVMAAIGLGFLTSSFRRHSWSSVAFNLFMLALGVQWAILLDGFLSQFPSGKVVITLFSIRLATMSAL
Mouse Rh 51 RNLTLMAALGFGFLSSSFRRHSWSSVAFNLFMLALGVQGTILLDHFLGQVLQWNKINNLSSIQIATMSTL
M3
M4
M5
3HD6 134 SVCVAFGAVLGKVSPIQLLIMTFFQVTLFAVNEFILLNLLKVKDAGGSMTIHTFGAYFGLTVTRILYRRN
Human RhAG 124 TVLISFGAVLGKTSPTQMLIMTILEIVFFAHNEYLVSEIFKASDIGASMTIHAFGAYFGLAVAGILYRSG
Mouse Rhag 134 TVLISFGAVLGKTSPIQMLIMTILEIAVFAGNEYLVTELFEASDTGASMTIHAFGAYFGLAVAGVLYRPG
Human RhD 122 SVLISVDAVLGKVNLAQLVVMVLVEVTALGNLRMVISNIFNTDYHMNMMHIYVFAAYFGLSVAWCLPKPL
Mouse Rh 121 PVLISAGAVLGKVNLVQLTVMVLMEAMAFGAIRFADEKVFKMTEHIIMMHGHVFGAYFGLTVAWWLSRSL
M6
M7
3HD6 204 L.....EQSKERQNSVYQSDLFAMIGTLFLWMYWPSFNSAISYHGDSQHRAAINTYCSLAACVLTSVAIS
Human RhAG 194 L.....RKGHENEESAYYSDLFAMIGTLFLWMFWPSFNSAIAEPGDKQCRAIVNTYFSLAACVLTAFAFS
Mouse Rhag 204 L.....RCEHPNDESVYHSDLFAMIGTLFLWIFWPSFNSAIADPGDHQYRAIVNTYMSLAACVITAYALS
Human RhD 192 P.....EGTEDKDQTATIPSLSAMLGALFLWMFWPSFNSALLRSPIERKNAVFNTYYAVAVSVVTAISGS
Mouse Rh 191 PRRVGENAQTEKVQMATSSSLFAMLGTLFLWIFWPAINSALLEGT.KKRNAVFNTYYALAVSAVTATSMS
M8
M9
3HD6 269 SALHKKGKLDMVHIQNATLAGGVAVGTAAEMMLMPYGALIIGFVCGIISTLGFVYLTPFLESRLHIQDTC
Human RhAG 259 SLVEHRGKLNMVHIQNATLAGGVAVGTCADMAIHPFGSMIIGSIAGMVSVLGYKFLTPLFTTKLRIHDTC
Mouse Rhag 269 SLVERRGRLDMVHIQNATLAGGVAVGTCADMEIPLYAAMTIGSIAGIISVLGYKFFSPLLANKLMIHDTC
Human RhD 257 SLAHPQGKISKTYVHSAVLAGGVAVGTSCHLIPSPWLAMVLGLVAGLISVGGAKYLPGCCNRVLGIPHSS
Mouse Rh 260 ALSHPQGKINMVHIHNAVLAGGVAVGAPGCLISSPWISMVLGLIAGLISIWGAKCPRACLNHML..QNSS
M10
M11
3HD6 339 GINNLHGIPGIIGGIVGAVTAASASLEVYGKEGLVHSFDFQGFNGDWTARTQGKFQIYGLLVTLAMALMG
Human RhAG 329 GVHNLHGLPGVVGGLAGIVAVAMG.....................ASNTSMAMQAAALGSSIGTAVVG
Mouse Rhag 339 GVHNLHGLPGVFGGLASIVAISWG.....................MSTASMAMQAAALGSSIGSAIVG
Human RhD 327 IMGYNFSLLGLLGEIIYIVLLVL...............DTVGAGNGMIGFQVLLSIGELSLAIVIALMS
Mouse Rh 328 GIHYTFGLPGLLGALTYYCLQIV...............TEPKSSDLWIITQTVTHIGALSFAVAMGMVT
M11
3HD6 409 GIIVGLILRLPFWGQPSDENCFEDAVYWEMPEGNS
Human RhAG 376 GLMTGLILKLPLWGQPSDQNCYDDSVYWKVPKTR.
Mouse Rhag 386 GLLTGLILKLPIWNQPPDEYCYDDSVSWKVPKFRE
Human RhD 381 GLLTGLLLNLKIWKAPHEAKYFDDQVFWKFPHLAV
Mouse Rh 382 GLLTGCLLSVRVWRAPHAAKYFDDQTFWEFPHLAV
